# Paraneoplastic Dermatomyositis as the Presenting Symptom of Tonsillar Squamous Cell Carcinoma: A Case Report

**DOI:** 10.7759/cureus.92974

**Published:** 2025-09-22

**Authors:** Max Vogel, Abigail Kammerer, Raj Dwivedi, Karyn Dyehouse, Terese Howes

**Affiliations:** 1 Oncology, University of South Carolina School of Medicine Greenville, Greenville, USA; 2 Radiation Oncology, University of South Carolina School of Medicine Greenville, Greenville, USA

**Keywords:** dermatomyositis, head and neck cancer, oropharyngeal cancer, paraneoplastic syndrome, tonsillar cancer

## Abstract

Dermatomyositis is an autoimmune multisystem disorder affecting skin and skeletal muscle that often presents with symmetric proximal muscle weakness and cutaneous manifestations such as rash. Dermatomyositis may present as a paraneoplastic phenomenon before, concurrent with, or after the diagnosis of cancer. Several cancers, including lung, breast, stomach, ovary, and kidney carcinoma, are frequently noted as the trigger for the development of paraneoplastic dermatomyositis. Less common, nasopharyngeal carcinomas have been associated with paraneoplastic dermatomyositis. Even more rare, we present a case of a 61-year-old male found to have dermatomyositis as the presenting symptom of p16-positive squamous cell carcinoma of the oropharynx involving the left tonsil with unilateral neck involvement and solitary lung metastasis. This case highlights a rare presentation of paraneoplastic dermatomyositis and emphasizes the importance of including p16+ tonsillar carcinoma in the differential diagnosis and screening protocols following the discovery of dermatomyositis.

## Introduction

Dermatomyositis is an autoimmune inflammatory disorder that impacts skin and skeletal muscle, usually presenting as progressive proximal muscle weakness and cutaneous manifestations such as rash [1]. Although dermatomyositis can occur as an isolated process, it often occurs as a paraneoplastic syndrome and acts as a harbinger of malignancy, often occurring before the actual diagnosis of cancer. Sources report up to 32% of cases of dermatomyositis may be associated with cancer [2]. Cancer risk is elevated for three to five years following diagnosis [3]. Dermatomyositis is most commonly associated with carcinomas of the lung, breast, stomach, ovary, and kidney. In rare instances, dermatomyositis can be associated with nasopharyngeal carcinoma. Paraneoplastic dermatomyositis is more common in patients of Southeast Asian and North African descent, although the frequency of this presentation is less than one in 1000 patients [1,2]. Few cases of dermatomyositis associated with primary tonsillar cancer exist in the literature. In this case, we present a patient with dermatomyositis subsequently diagnosed with p16-positive squamous cell carcinoma of the left tonsil.

## Case presentation

**Figure 1:**
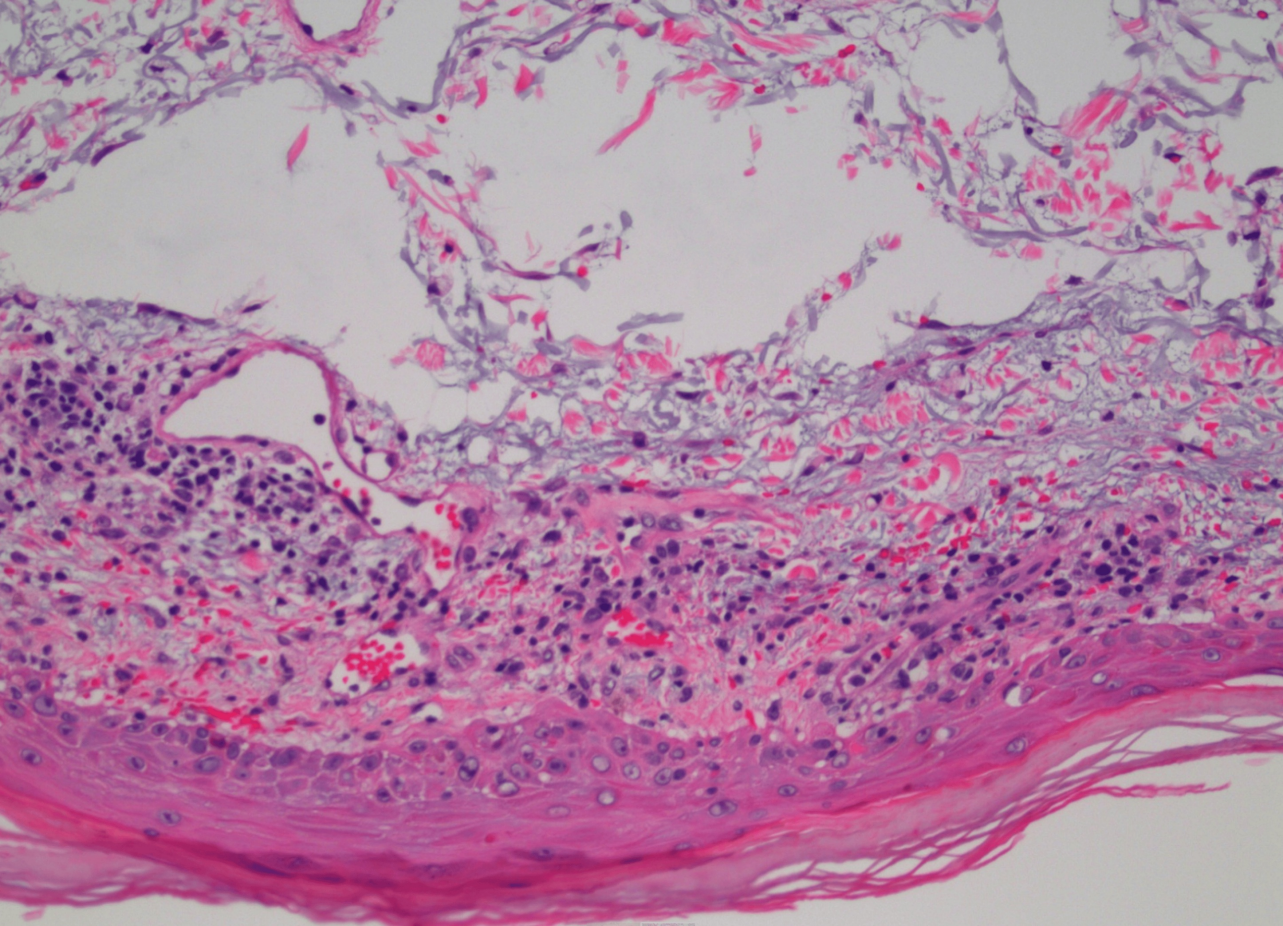
Hematoxylin and eosin staining of skin punch biopsy demonstrating interface dermatitis.

**Figure 2:**
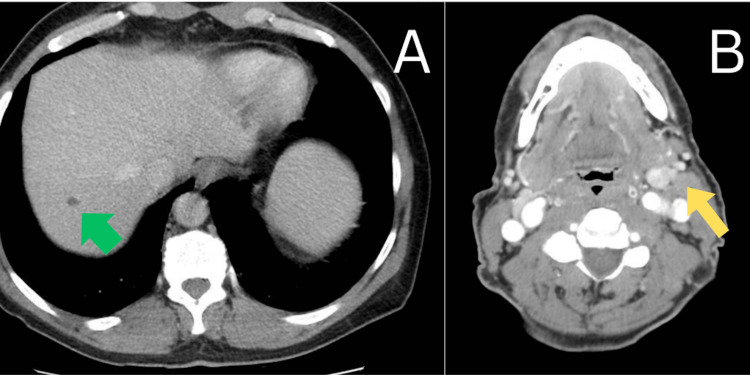
CT scan of the abdomen, pelvis, and neck with contrast. Image A demonstrates one of several indeterminate hypoattenuating lesions in the liver (green arrow). Image B demonstrates an enlarged, 13-mm, level two submandibular lymph node (yellow arrow).

**Figure 3:**
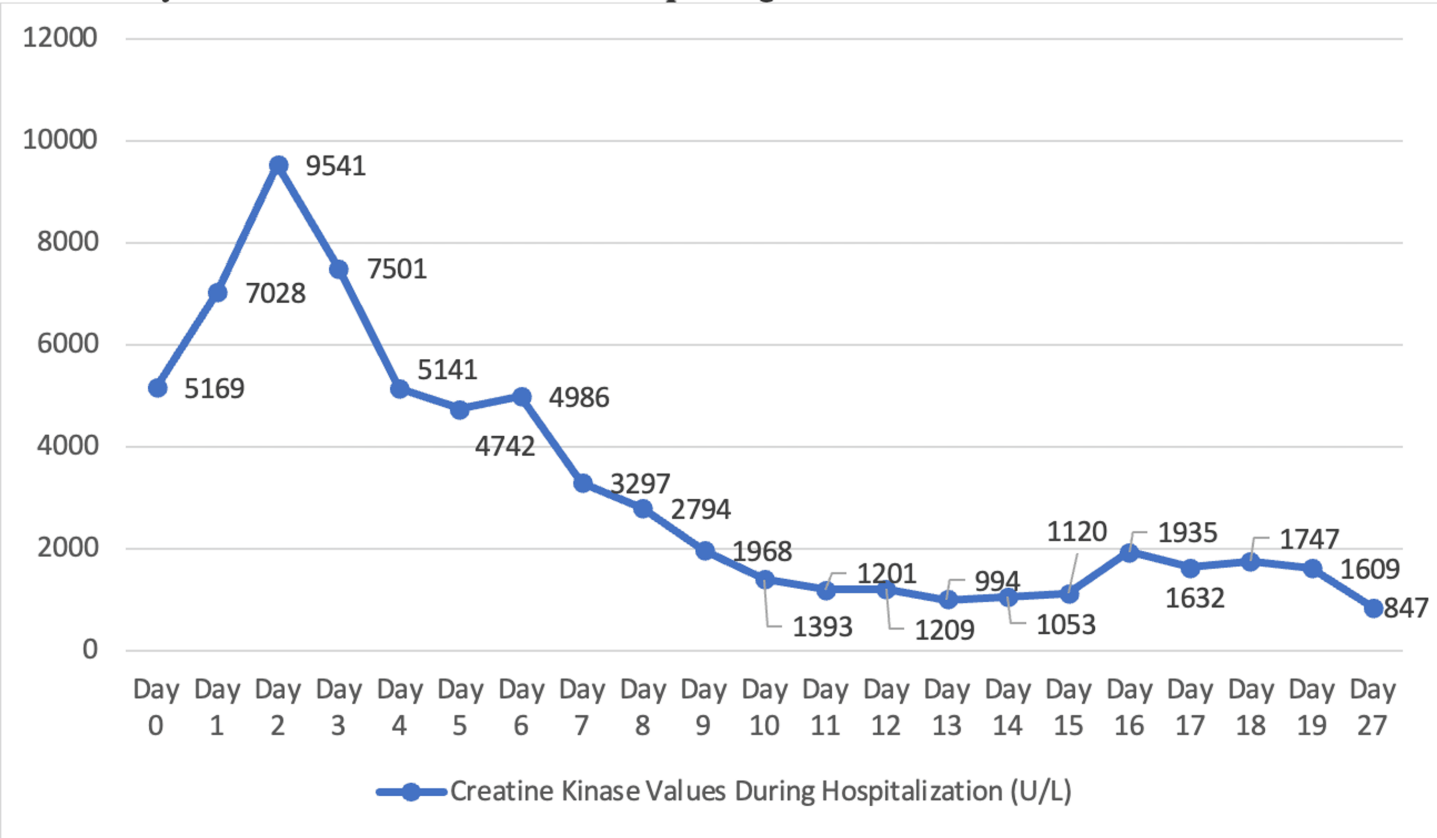
Creatine kinase values from hospital admission to discharge. Note: reference range = 41-331 U/L.

A 61-year-old male presented to his primary care physician in 2018, several weeks after noticing a swollen left submandibular lymph node, a mild rash on the chest and bilateral upper arms, and bilateral shoulder pain. His primary care provider prescribed triamcinolone cream for the rash, and a CT scan of the neck, complete blood count, and comprehensive metabolic panel to work up his symptoms. One week later, he returned to his primary care provider with worsening of the rash, which spread to his lower legs and ankles and began to scale. His shoulder pain progressed to weakness and was accompanied by significant fatigue. At this time, his submandibular lymphadenopathy was persistent but unchanged. The patient elected to delay his neck CT due to an upcoming vacation. Prior lab work-up was unremarkable. He was started on prednisone and underwent a punch biopsy of the right anterior thigh. Pathology demonstrated interface dermatitis consistent with dermatomyositis (Figure 1). The patient returned to his primary care provider one month later and was scheduled for a CT of the abdomen, pelvis, and neck to rule out malignancy. Imaging demonstrated a known enlarged level two submandibular lymph node measuring 13 millimeters and several small low attenuation lesions in the liver, but was otherwise unremarkable (Figure 2). Creatine kinase was obtained and was elevated at 472 U/L. He was started on prednisone but discontinued after two weeks due to unspecified adverse effects. The patient was referred to rheumatology, but declined the referral. Two months later, he presented to the emergency department with shortness of breath, worsening muscle weakness and rash, and difficulty swallowing. He was admitted for further workup. Creatine kinase was 5169 U/L on admission, indicating rhabdomyolysis in the setting of dermatomyositis. During his hospital stay, he was started on a course of 20 milligrams of prednisone three times per day, which was eventually switched to Solu-Medrol 60 milligrams three times per day due to worsening muscle weakness and dysphagia. He underwent esophagogastroduodenoscopy during hospitalization to rule out anatomical causes of dysphagia. He was found to have a stricture, which was dilated. Despite corticosteroids, his weakness and dysphagia continued to worsen, and he was transferred to a second hospital after one week due to escalating care needs. He was seen by rheumatology, who recommended high-dose IV corticosteroids. Per recommendations by rheumatology and hematology, the patient was started on a three-day course of intravenous immunoglobulin therapy. One day after transfer, he developed shortness of breath, bradycardia, and hypoxia with oxygen saturations as low as 82%. After two days of continued clinical deterioration, he was intubated. The patient developed hemolytic anemia and thrombocytopenia, suspected to be secondary to dermatomyositis. Five days after admission, hematology recommended initiation of rituximab. Intubation was complicated by suspected aspiration pneumonia, requiring an eight-day course of vancomycin and piperacillin/tazobactam. The patient was successfully extubated five days after intubation and was recommended for tracheostomy and percutaneous endoscopic gastrostomy tube placement. Around one month after admission, cell counts and creatine kinase reached near normal values, and the patient was deemed stable for discharge to a long-term acute care facility, with plans for intravenous immunoglobulin two times monthly for six months and 60 milligrams of prednisone to continue in the outpatient setting. Creatine kinase trends during hospitalization are presented in Figure 3. After two weeks in long-term acute care, the patient was discharged and began follow-up with rheumatology in the outpatient setting. His weakness continued to improve, and the patient was started on mycophenolate mofetil around three months after discharge as he was gradually weaned off corticosteroids. Around eight months after hospital discharge, the patient was able to discontinue corticosteroids while remaining on maintenance mycophenolate mofetil.

**Figure 4:**
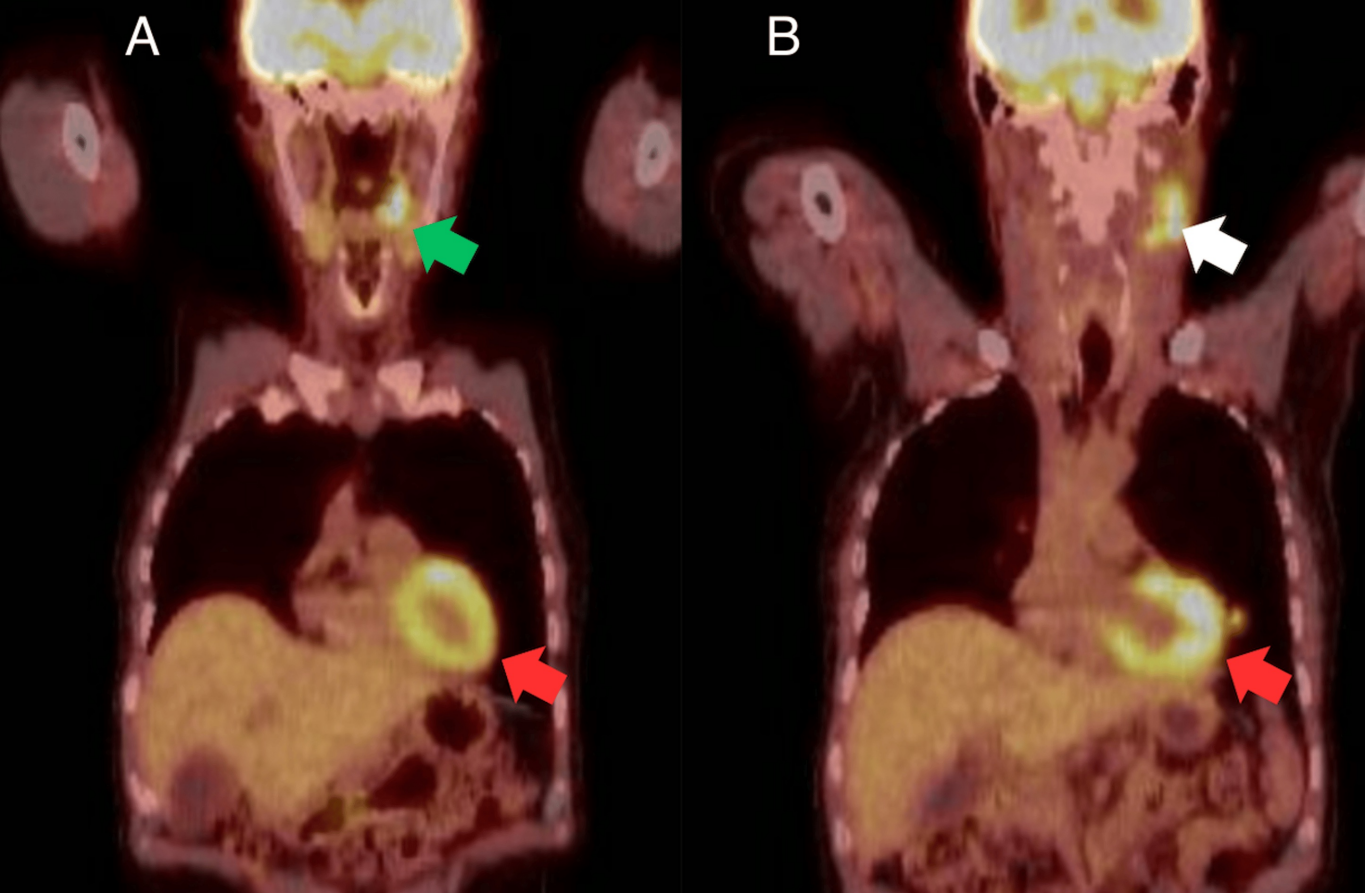
PET/CT demonstrating left peritonsillar, left upper jugular lymph node chain, and left lingula nodule hyperactivity. Image A demonstrates left peritonsillar (green arrow) and left lingula (red arrow) hyperactivity. Image B demonstrates the left upper jugular lymph node chain (white arrow) and left lingula (red arrow) hyperactivity.

**Figure 5:**
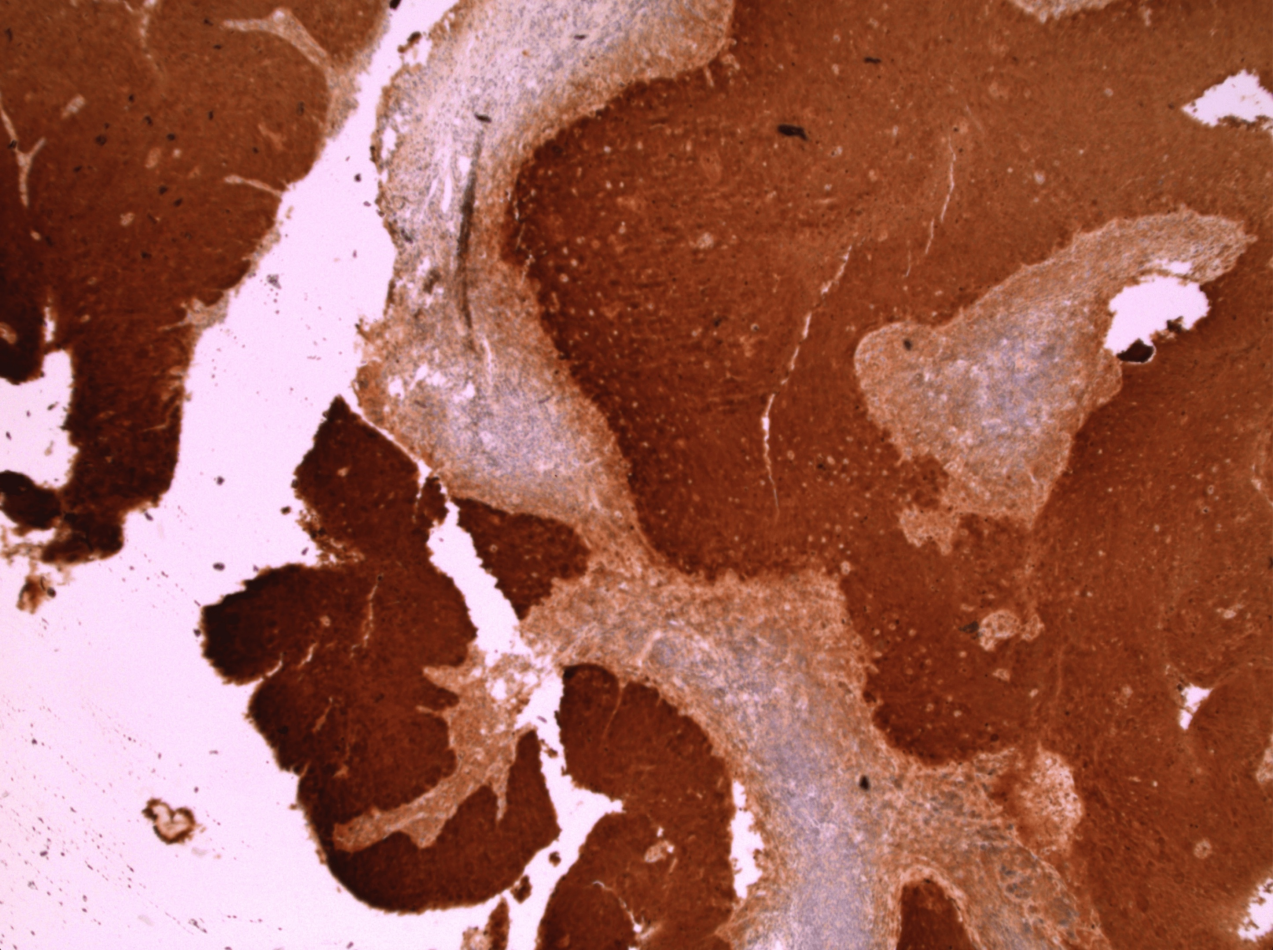
Left tonsillar biopsy demonstrating p16-positive, poorly differentiated squamous cell carcinoma.

**Figure 6:**
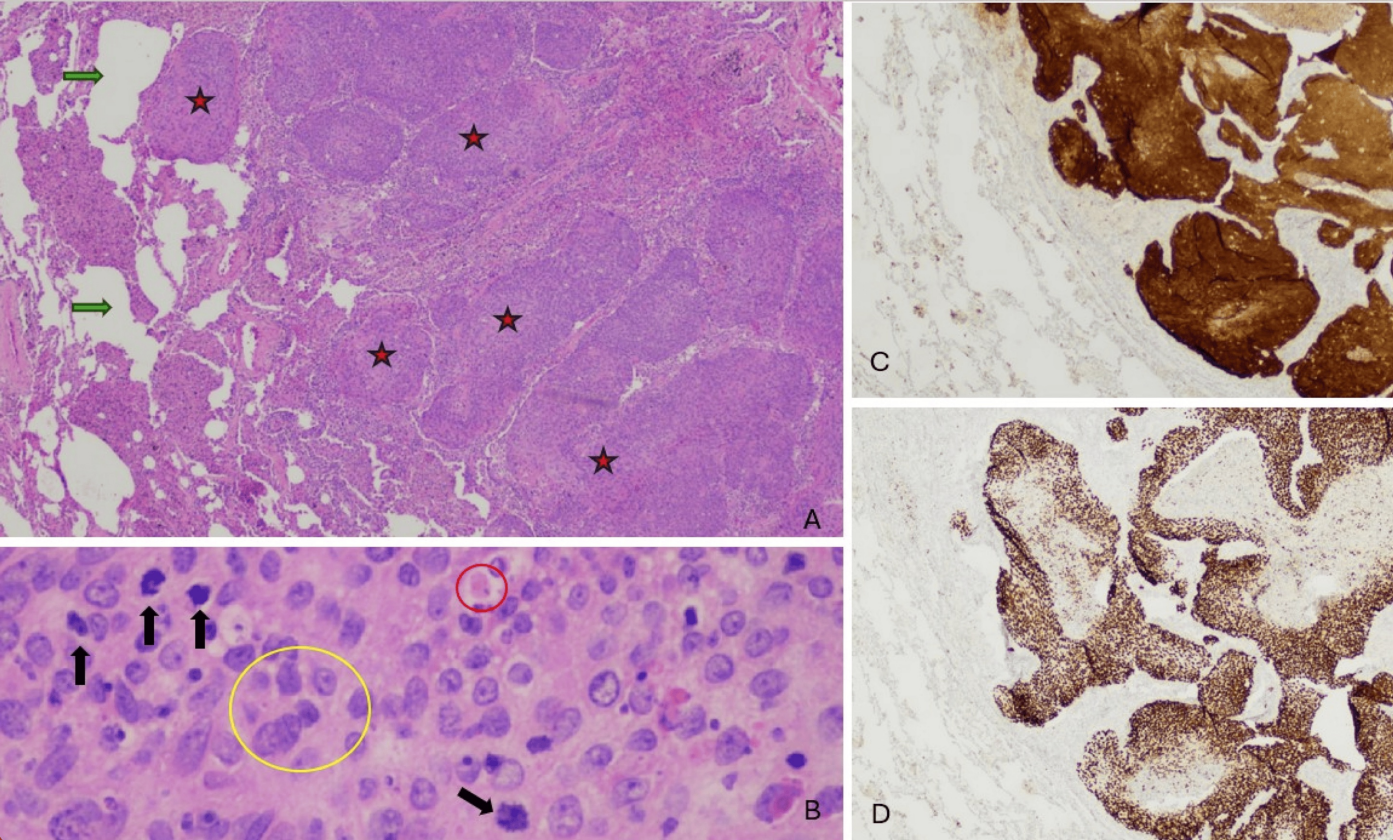
Histology of left lingula biopsy. Sections from the patient's left upper lobe wedge resection showed atypical nonkeratinizing cells in a sheet-like architecture invading into the subpleural alveolated lung parenchyma (image A, red stars). Closer inspection revealed atypical cells with irregular nuclear contours, nuclear anisocytosis, and pleomorphism (image B, yellow circle). Scattered apoptotic cells were present (image B, red circle), and mitotic figures were abundant (image B, black arrows). P16 and p40 immunohistochemistry were performed. P16 revealed diffuse block-like reactivity in malignant cells (image C), and p40 showed diffuse nuclear reactivity in malignant cells (image D), confirming squamous differentiation.

Just prior to discontinuing corticosteroids, the patient’s rheumatologist ordered tuberculosis testing due to his prolonged course of immunosuppressive therapy. His initial Tuberculosis Gold test was indeterminate, prompting a chest X-ray and T-spot testing. Chest X-ray revealed a new left lobe nodularity, prompting a full-body PET scan. PET scan revealed left peritonsillar, left upper jugular lymph node chain, and left lingula nodule hyperactivity (Figure 4). The PET/CT imaging was performed one year after the initial presentation of dermatomyositis symptoms. The patient was referred to otolaryngology and underwent tonsillar biopsy, revealing a p16-positive, poorly differentiated squamous cell carcinoma of the left tonsil (Figure 5). Less than one month after diagnosis of tonsillar carcinoma, the patient underwent video-assisted thoracoscopy with wedge resection and mediastinal lymph node biopsy to characterize the lymphadenopathy and pulmonary nodule noted on prior PET scan. Biopsied lymph nodes were negative for tumor, with pathology of the lung nodule revealing p16-positive squamous cell carcinoma consistent with metastatic disease excised with negative margins (Figure 6). The patient was staged as cT2, cN1, cM1a (IV), p16-positive squamous cell carcinoma of the left tonsil, unilateral neck node involvement, and solitary lung metastasis. The multidisciplinary recommendation was to proceed with definitive treatment of his tonsil cancer, given a solitary site of metastasis, which had been resected. Approximately three weeks after wedge resection, the patient began definitive chemoradiation consisting of 70 Gray in 35 fractions to the left tonsil and unilateral neck concurrent with three cycles of cisplatin (100 mg/m^2^ intravenous every 21 days). The patient tolerated treatment well and remains under surveillance with CT scans of the neck and chest initially every six months with yearly fluorodeoxyglucose (FDG)-PET/CT, now followed clinically without a plan for additional imaging. The patient is now five years from completion of therapy with no evidence of disease, and he no longer requires immunosuppressive therapy for dermatomyositis, with no flares since completing treatment for his cancer.

## Discussion

Paraneoplastic dermatomyositis is a well-documented phenomenon, but its pathogenesis is poorly understood. Definitive diagnosis requires a muscle biopsy and is supported by elevated creatine kinase [4]. Several underlying mechanisms have been suggested to potentially explain paraneoplastic dermatomyositis. One theory suggests a hormonal basis for paraneoplastic dermatomyositis, in which tumor-produced hormones trigger a lymphocyte-mediated autoimmune response [2,5]. Other theories suggest molecular mimicry as the driving process, in which tumor-produced antigens result in the production of autoantibodies. Certain antibodies have been found to have high predictive value for predicting cancer in patients diagnosed with dermatomyositis. Anti-TIF1γ antibody, first described in 2006, has been shown to have a 78% sensitivity and 89% specificity for predicting a subsequent cancer diagnosis [4]. TIFIγ is a protein that has been shown to function as a tumor suppressor in certain cancer types, including renal cell carcinoma, while overexpression is thought to contribute to the formation of breast and colorectal cancer [4]. The International Myositis Assessment and Clinical Studies Group has published guidelines for cancer screening in patients with dermatomyositis. Several autoantibodies, including anti-TIF1γ, are used in their methodology to risk-stratify patients. While an autoantibody panel is often included in the workup for suspected dermatomyositis, it was not performed in the workup of the patient presented in this case [6]. Although the patient did undergo initial CT imaging to screen for cancer, the lack of autoantibody panel results makes the patient's risk for developing malignancy unclear. Had the patient been documented positive for high-risk autoantibodies like anti-TIF1γ, he may have undergone more robust screening that would have led to earlier detection of his underlying malignancy. Paraneoplastic dermatomyositis associated with tonsillar carcinoma has been documented in the literature, but cases are exceedingly rare. Current guidelines for screening do not include workup for the identification and diagnosis of potential tonsillar or other nasopharyngeal carcinoma [6]. Our literature review returned only four cases of dermatomyositis associated with tonsillar carcinoma. Two cases have been documented in South Korea [3]. In Caucasian patients, one report exists of a documented case in Italy and another in the United States [7,8]. This case represents only the second documented case of paraneoplastic dermatomyositis associated with tonsillar carcinoma in North America. In each documented case, the occurrence of dermatomyositis preceded the patient’s cancer diagnosis by several months to a year. Treatment of paraneoplastic dermatomyositis requires immunosuppressive therapy and treatment of the underlying malignancy. Similar to the patient presented in this case, patients in previously documented cases had resolution of symptoms following immunosuppression and definitive treatment of their cancer [3,7,8]. The implications of dermatomyositis on the prognosis of tonsillar carcinoma have not been firmly established. In two prior cases, patients had recurrent metastatic disease within 15 months of treatment. Long-term follow-up was not documented in the other two cases [3,7,8].

## Conclusions

Although rare, dermatomyositis may be the presenting symptom in patients diagnosed with tonsillar carcinoma. Current published guidelines do not recommend routine screening for head and neck cancers in patients diagnosed with dermatomyositis. The patient presented in this case was diagnosed with tonsillar carcinoma based on an incidental finding on chest X-ray and did not receive routine cancer screening between his diagnosis of dermatomyositis and the discovery of his cancer. Documentation of cancer risk was also limited by the lack of an autoantibody panel at the time of diagnosis with dermatomyositis. Consideration of autoantibody screens can guide providers in assessing risk and appropriate cancer screening after dermatomyositis diagnosis. Despite its rarity, tonsillar and nasopharyngeal carcinomas should not be ruled out as a potential trigger of dermatomyositis. Diagnostic tools, including routine PET, direct visualization, and endoscopy, could be considered in routine monitoring after diagnosis, especially in patients with severe or recurrent dermatomyositis with otherwise negative malignancy workup.
